# Homocysteine as a Predictor Tool in Multiple Sclerosis

**DOI:** 10.15190/d.2021.14

**Published:** 2021-09-28

**Authors:** Radu Razvan Mititelu, Carmen Valeria Albu, Manuela Violeta Bacanoiu, Vlad Padureanu, Rodica Padureanu, Gabriela Olaru, Ana-Maria Buga, Maria Balasoiu

**Affiliations:** ^1^Department of Microbiology, University of Medicine and Pharmacy of Craiova, Romania; ^2^Department of Neurology, University of Medicine and Pharmacy of Craiova, Romania; ^3^Department of Sports and Kinetic Therapy, University of Craiova, Romania; ^4^Department of Internal Medicine, University of Medicine and Pharmacy of Craiova, Romania; ^5^Department of Biochemistry, University of Medicine and Pharmacy of Craiova, Romania

**Keywords:** multiple sclerosis, vitamin status, homocysteine, cobalamin.

## Abstract

Multiple sclerosis (MS) is a progressive and irreversible disease which affects the central nervous system (CNS) with still unknown etiology. Our study aimes to establish the homocysteine pattern that can predict the MS diseases progression and to identify a potential disease progression marker that can be easy to perform and non-invasive, in order to predict the diseases outcome. In order to achieve this goal, we included 10 adult RRMS subjects, 10 adult SPMS subjects and 10 age-matched healthy subjects. The homocysteine plasma level was measured using automated latex enhanced immunoassay and the cobalamin and folate measurements were performed using automated chemiluminescence immunoassay (CLIA). HCR was calculated by dividing the homocysteine plasma level by cobalamin plasma level. We found that the homocysteine level in plasma of both RRMS patients and SPMS group are significantly increased compared with the control group. There is a significantly higher concentration of homocysteine in SPMS group compared with the RRMS group. In addition, the HCR is significantly increased in SPMS compared with the RRMS group and is a very good index of disease severity.

## INTRODUCTION

Multiple sclerosis (MS) is a progressive and irreversible disease which affects the central nervous system (CNS) and was often followed by neurological dysfunction and difficult to predict^1,2^. More than two million young individuals worldwide were affected. The etiology of MS is still unclear, and the pathogenesis has not been well defined yet. A personalized therapy is more effective, but it is limited due to the absence of specific biomarkers and treatment algorithms^3,4^. However, in order to evaluate the treatment response and to identify the right moment for therapeutic switch we need to establish a complex profile of patients that include environmental factors, demographic factors, imaging and diseases progression biomarkers^3-5^.

Complex interactions between environmental and genetic factors are involved in the autoimmune inflammatory process^6^. The relationship between the vitamin status and the immune system is bidirectional. Here are still questions to be addressed, one of these questions is that if the neurodegenerative diseases progression is due to nutritional and vitamin deficit or these deficits are neurodegenerative-related. Studies before showed that the hyperhomocisteinemia is an independent risk factor for many neurodegenerative diseases such as dementia or neuromyelitis optica spectrum disorders (NMOSD) and the homocysteine level is associated with an increased relapse risk or poor functional outcome^7,8^.

Vitamin B12, homocysteine and folate have been possible participants in the neurodegenerative process of many chronic diseases including MS. Homocysteine is a neurotoxic amino acid that accumulates in patients with neurodegenerative disorders. Homocysteine exerts direct effects on activation of macrophages and cell damage with a key role in human health. Increased homocysteine levels play an underestimated role in the process of many neurological diseases like MS^9^. Vitamin B12 and folate are needed in the process of methionine-synthase mediating the conversion of homocysteine to methionine^10^. Essential factors for methionine synthesis of homocysteine are 5-methyltetrahydro-folate and Vitamin-B12^11^. An increased level of homocysteine may be caused by folate and vitamin B12 deficiency. However, divergent results of the relation between folate, Vitamin B12 and homocysteine are reported. A meta-analysis showed lower Vitamin B12 levels in patients with MS, increased homocysteine levels and no significant difference in folate noted between controls and MS groups^12-14^. Studies before showed that MS patients had higher levels of homocysteine which were associated with disease progression, but other studies didn’t confirm this hypothesis^15,16^.

Increased serum homocysteine levels may be a risk factor for neurological decline in MS and may have a neurodegenerative effect^17,18^. Not all the studies have demonstrated a relationship between MS progression and elevated serum homocysteine^19,20^. Metaanalysis studies failed to take some critical factors into account including age, sex, disease severity and/or phase, and/or race of study population. Evidence show that serum levels of cobalamin are decreased in relapsing-remitting patients in comparison with control group. Other study has also shown that some patients with multiple sclerosis, as a sign of B12 deficiency, are also suffering from comorbidities like macrocytosis or megaloblastic anemia^21^. Vitamin B12 is an important cofactor in the formation of myelin sheath^22^. It has a role in the modulation of the immune system (cytokines TNF-gamma activity) so the cobalamin deficiency may worsen the inflammation that might be seen in MS^23^. Multiple treatments with cobalamin have been reported with decreased relapse risk or improvements in EDSS scores^24,25^.

In this study, our hypothesis is that the vitamin status can affect in a controllable fashion the homocysteine level in plasma. This can be a key factor in the management of MS patients in order to control the modifiable risk factor and limit diseases progression. A second hypothesis is that the homocysteine/ cobalamin ratio can be a useful tool for monitoring the diseases progression status.

## Materials and Methods

A total of 30 adult subjects manifesting both RRMS (n=10) and SPMS (n=10), as well as 10 heathy volunteers participated in the present study. The inclusion criteria were the age (>18 years old) and no other health problems besides MS. The disease severity was established in the study using Expanded Disability Status Scale (EDSS). All the MS patients included in this study were treated with standard therapy for MS. The study was conducted according to the guidelines of the Declaration of Helsinki and approved by the Institutional Review Board of the University of Medicine and Pharmacy of Craiova (Registration No. 96/2019). Informed consent was obtained from all the subjects involved in the study.

### Sample Collection

The blood samples were collected in the morning, after overnight fasting. One sample was collected on commercially available EDTA (ethylene-diamine-tetraacetic acid) tubes and was used to perform complete blood counting (CBC). For plasma collection, the blood sample were collected on sodium citrate commercially available tubes. Blood cells were immediately separated from plasma by centrifugation at 1000 g for 10 minutes in a refrigerated centrifuge (5417R Eppendorf). The blood cells were removed from plasma immediately following centrifugation and 0.5 ml plasma aliquot were stored at -80°C for further analysis of homocysteine. After blood cells separation, homocysteine is stable in plasma for 3 months at -20°C. For cobalamin and folate measurements, venous blood samples were collected on vacutainers from Becton Dickinson (BD) and the blood was allowed to clot for 20 minutes at room temperature (RT). The clot was then removed by centrifugation at 2000 g for 10 minutes in a refrigerated centrifuge (5417R Eppendorf) and the serum sample was used for analysis.

### Complete blood counting (CBC)

 To perform complete blood counting (CBC) we have used 5-part differential cell counter that use both flow-cytometry and Coulter¢s principle to differentiate all the five types of peripheral blood cells (neutrophils, monocytes, lymphocytes, basophils and platelets). *Plasma homocysteine measurement* This was performed using an automated latex enhanced immunoassay for the quantitative measurement of total L-Homocysteine in human plasma using ACL TOP 500 (Instrumentation Laboratory Company, U.S.A), according to the manufacturer instructions.

###  Cobalamin and folate measurements

These were performed using an automated chemiluminescence immunoassay (CLIA) for the quantitative measurement of plasma cobalamin that use the acridinium tracer and paramagnetic microparticles (Arhitect i1000SR, Abbott Company). All the measurements were performed according to the manufacturer instructions. Homocysteine/Cobalamin ratio (HCR) was calculated by dividing the homocysteine plasma level by cobalamin plasma level.

###  Statistical analysis

 The data was analyzed using the GraphPad Prism 5.0 software. The results were calculated as mean ± standard deviation (SD). The significant differences between the studied groups for normally distributed data were analyzed by One-way Anova test. Non-parametric with a receiver operating characteristic (ROC) curve for sensitivity and specificity with area under the ROC curve (AUC) for accuracy of the tests. P values less than 0,05 (p≤0,05) were established as cut-off for significant changes.

## Results

We included 20 adult MS subjects, according to McDonald criteria^23^ and 10 aged-matched healthy subjects, without history of an auto-immune or inflammatory disease. Demographic and clinical characteristics are presented in Table 1. 

**Table 1:** Demographic and clinical characteristics of MS subjects (mean±SD)

	Healthy group	RRMS group	SPMS group
Age	37.0±11.0	39.69±5.97	47.49±12.7
EDSS	NA	2.81(1-4)	4.93 (2-6)
Hgb (g/dl)	14.0±1.4	13.56±0.62	13.70±1.32
MCV	80.2±0.6	89.25±4.02	88.91±2.43
MCH	27.0±0.7	34.00±4.02	35.00±4.02
RDW	12.0±1.8	13.90±1.44	14.00±1.41

**Figure 1:**
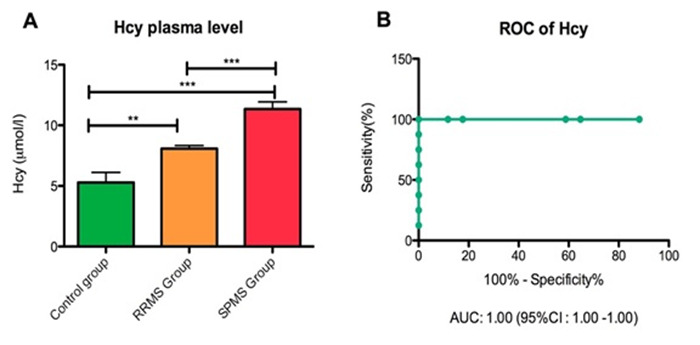
Pattern of homocysteine test in plasma (**A**) Homocysteine level in plasma in all groups (*** p=0.0001; ** p=0.01); (**B**) ROC analysis of homocysteine.

**Figure 2:**
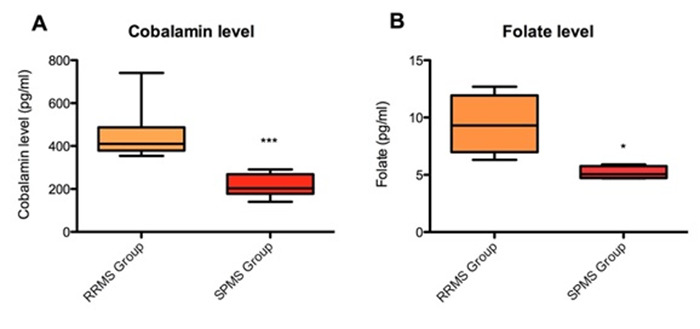
Pattern of vitamin status in plasma (**A**) Cobalamin level in plasma in MS groups (*** p=0.0001); (**B**) Folate level in plasma in MS groups (* p=0.02).

**Figure 3:**
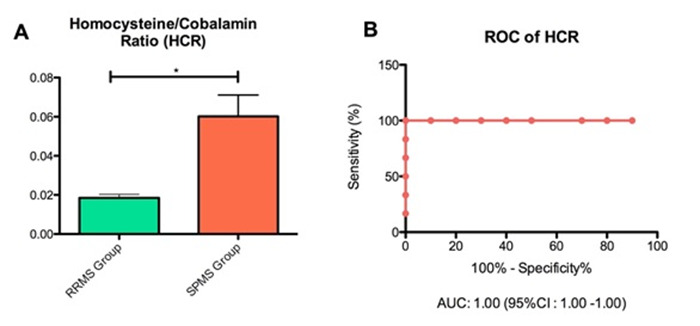
Pattern of homocysteine/cobalamin ratio (**A**) HCR in RRMS vs SPMS group (* p=0.02); (**B**) ROC analysis of HCR.

We measured homocysteine level in plasma of both RRMS and SPMS patients. Our results showed that here is a significant difference between RRMS and SPMS groups (p<0.05) and between MS groups and the control group (p<0.05) (Figure 1). To appreciate the vitamin status that is related to homocysteine metabolic pathway, we measured the cobalamin and the folate level in plasma. We found that both the cobalamin and folate level is decreased in SPMS group compared with RRMS group (p<0.05) (Figure 2). We found that the homocysteine/cobalamin ratio (HCR) is significantly increased in SPMS group compared with RRMS group (Figure 3).

## Discussion

Homocysteine is a sulphur containing compound that results from the methionine metabolic pathway and was described as a key factor in many diseases including MS. In the human body, the homocysteine metabolic pathway is linked to cobalamin and folate level. These active compounds act as a coenzyme for the enzyme that catalyzes the demethylation reaction of homocysteine^26^. Deficiency of these cofactors can lead to an increased homocysteine level and an increased risk of poor outcome in many autoimmune diseases, such as MS^27-30^.

Studies before showed that the homocysteine is one of the key risk factors for endothelial dysfunction that can be modified^31,32^. The homocysteine level in plasma was strongly correlated with the cognitive dysfunction due to vascular lesion^32^. In the last decade, the research focus was on the molecular mechanism of hyperhomocysteinemia in auto-immune and neurodegenerative diseases. In MS patients, it was described a significant increase in homocysteine level in plasma compared with healthy control, but the mechanism of action is still not fully understood^33^.

Despite of accumulating evidence that prove the homocysteine role in MS diseases progression, only a few studies were focused on homocysteine level pattern in RRMS compared with SPMS patients^34^. In this light, our study was focused on establishing the homocysteine pattern that can predict the MS diseases progression and to identify a potential disease progression marker that can be easy to perform and non-invasive, in order to predict the diseases outcome. In this study we found that the homocysteine level in plasma of both RRMS patients and SPMS group are significantly increased compared with the control group. Also, there is a significant higher concentration of homocysteine in SPMS group compared with RRMS group (Figure 1 A,B). We showed that the homocysteine plasma level is a feasible marker for disease status with AUC of 1.00 (95% CI of 1.00) according with ROC analysis.

In order to identify a direct correlation of Hyperhomocysteinemia with vitamin status, we measured the cobalamin and folate plasma level. Since the control group has an unchanged homocysteine level, we did not include this for HCR calculation. We found a significantly decreased concentration in cobalamin and folate plasma level in SPMS patients compared with the RRMS patients (Figure 2). Here we showed an opposite pattern of cobalamin and folate availability with homocysteine plasma concentration. Also, we established that the homocysteine and cobalamin ratio is significantly increased in SPMS group compared with RRMS group and is a very good index of disease severity with AUC of 1.00 ((95% CI of 1.00) according with ROC analysis. However, here is still a lack of evidence regarding the molecular mechanism involved in disease progression regarding homocysteine metabolic pathway.

###  Limitation of the study

Our study is limited due to the small number of participants. Further studies on a large cohort are needed in order to validate these results and to establish the right time for intervention and the right pharmacological approach for MS patients.

## Conclusions

Our study showed that the vitamin status is a key factor in the metabolic control of hyper-homocysteinemia in MS patients. For the first time we showed that the homocysteine/cobalamin ratio can be an important index for disease severity that is easy to perform and non-invasive that should be taken into account in the management of MS patients. However, further studies are necessary in order to establish an effective multimodal approach treatment that can modify the important risk factors for diseases progression.

## Key points

**◊** Vitamin status can affect in a controllable fashion the homocysteine level in plasma, thus controlling the modifiable risk factor and limit diseases progression.

**◊** Homocysteine/cobalamin ratio can be a useful tool for monitoring the diseases progression status.
